# Curcumin in Combination with Aerobic Exercise Improves Follicular Dysfunction via Inhibition of the Hyperandrogen-Induced IRE1*α*/XBP1 Endoplasmic Reticulum Stress Pathway in PCOS-Like Rats

**DOI:** 10.1155/2021/7382900

**Published:** 2021-12-26

**Authors:** Yaling Zhang, Yajing Weng, Daojuan Wang, Rong Wang, Lihui Wang, Jianjun Zhou, Shanmei Shen, Hongwei Wang, Yong Wang

**Affiliations:** ^1^State Key Laboratory of Analytical Chemistry for Life Science & Jiangsu Key Laboratory of Molecular Medicine, Medical School, Nanjing University, Nanjing 210093, China; ^2^Danyang Hospital Affiliated to Nantong University, Danyang, Jiangsu 212300, China; ^3^Department of Endocrinology, The Affiliated Drum Tower Hospital, Medical School, Nanjing University, Nanjing 210093, China

## Abstract

Combining diet with exercise can improve health and performance. Exercise can reduce androgen excess and insulin resistance (IR) in polycystic ovary syndrome (PCOS) patients. Curcumin is also presumed to improve the follicle development disorder. Here, we investigated the effects of a combination therapy of oral intake of curcumin and exercise on hyperandrogen-induced endoplasmic reticulum (ER) stress and ovarian granulosa cell (GC) apoptosis in rats with PCOS. We generated a PCOS model via continuous dehydroepiandrosterone subcutaneous injection into the necks of Sprague Dawley rats for 35 days. PCOS-like rats then received curcumin treatment combined with aerobic (treadmill) exercise for 8 weeks. We found that compared to control rats, the ovarian tissue and ovarian GCs of hyperandrogen-induced PCOS rats showed increased levels of ER stress-related genes and proteins. Hyperandrogen-induced ovarian GC apoptosis, which was mediated by excessive ER stress and unfolded protein response (UPR) activation, could cause follicle development disorders. Both curcumin gavage and aerobic exercise improved ovarian function via inhibiting the hyperandrogen-activated ER stress IRE1*α*-XBP1 pathway. Dihydrotestosterone- (DHT-) induced ER stress was mitigated by curcumin/irisin or 4*μ*8C (an ER stress inhibitor) in primary GC culture. In this in vitro model, the strongly expressed follicular development-related genes *Ar*, *Cyp11α1*, and *Cyp19α1* were also downregulated.

## 1. Introduction

Polycystic ovary syndrome (PCOS), a common disease caused by complex endocrine and metabolic abnormalities among women of reproductive age, is characterized by hyperandrogenism, ovulatory dysfunction, and formation of polycystic ovaries. Obesity, low-grade chronic inflammatory status, and insulin resistance (IR) often coexist in PCOS. Its prevalence ranges from 8 to 13% [1, 2]. The clinical manifestations and biochemical characteristics of PCOS are highly heterogeneous. The increased leutinizing/follicle-stimulating hormone (LH/FSH) ratio, hyperandrogenemia, and hyperinsulinemia are the major endocrine characteristics of PCOS [3, 4]. In addition, sparse or anovulation is an important feature of PCOS. Follicular development is characterized by follicular arrest, the emergence of nondominant follicles, and/or persistent nonovulation. However, the primary causes of ovarian dysfunction and ovulatory disruption in PCOS are incompletely clear. Most studies report that hyperandrogenism is the main cause of infertility owing to PCOS. Our previous research showed that hyperandrogen dramatically augments oxidative stress (OS) and fibrotic factor activation in the ovary [5] and stimulates chronic low-grade inflammation in the ovary to activate the NOD-like receptor family pyrin domain-containing 3 (NLRP3) inflammasome, further inducing ovarian granulosa cell (GC) pyroptotic death, follicular dysfunction, and ovarian interstitial cell fibrosis [6]. GCs may undergo excessive OS, chronic low-grade inflammation, and increased apoptosis [7–9]. GC growth and differentiation are key to the initiation and growth of primordial follicles, and GC insufficiency contributes to abnormal follicular development.

The endoplasmic reticulum (ER), an intracellular organelle, controls calcium homeostasis, lipid synthesis, and protein folding, posttranslational modification, and trafficking. OS, hypoxia, and overexpression of normal and/or misfolded proteins can lead to ER stress. To maintain ER homeostasis, the unfolded protein response (UPR) is triggered. The UPR is induced by stress sensors on the ER membrane, namely, activating transcription factor 6 (ATF-6), double-stranded-RNA-dependent protein kinase- (PKR-) like ER kinase (PERK), and inositol-requiring protein 1*α* (IRE1*α*). Among these, IRE1*α* can be activated upon ER stress. Activated IRE1 induces the splicing of X-box-binding protein 1 (XBP1) mRNA by cleaving off its intron. Activated IRE1*α* triggers unconventional cytoplasmic splicing of XBP1 mRNA. IRE1*α* excises a 26-nucleotide fragment from the XBP1 mRNA under ER stress to generate a spliced version encoding the functionally active XBP1 protein, which mediates adaptation to ER stress by inducing genes involved in protein folding and quality control [10]. Subsequently, cells undergo various effective processes to adapt to and prevent ER stress-induced apoptosis [11–13]. Recent research showed that ER stress is activated not only in cumulus cells from PCOS patients, but also in GCs and cumulus-oocyte complexes (COCs), affecting oocyte maturation, follicle formation, and ovulation [14]. We speculate that long-term, continuous hyperandrogen exposure causes ER stress and induces GC autophagy or apoptosis. This can lead to the accumulation of small follicles around the ovary, polycystic morphology, and damage to follicular maturation and anovulation follicular development disorders in PCOS.

Due to the complex pathogenesis of PCOS, the main treatment is aimed at correcting the endocrine disorders of PCOS patients, improving their clinical symptoms, and preventing the occurrence of long-term complications. Lifestyle changes, including diet and exercise, are the first-line treatments for PCOS. There are many clinical studies have provided strong evidence of strategic relevance of dietary management in the treatment of women with PCOS. For example, Mediterranean diet, low-carb diet, and supplementary antioxidant diet all have anti-inflammatory, antioxidant, antiobesity, and anti-IR effects on PCOS [15, 16]. Among the auxiliary antioxidant factors, curcumin has attracted much attention in recent years. Curcumin is a natural medicine containing phenol and quinone groups extracted from turmeric in the ginger family. It is well known as a safe dietary supplement worldwide [17, 18]. In several randomized, double-blinded, clinical trial, patients with PCOS received curcumin (in doses of 500-1500 mg 3 times daily for 1-3 months) or placebo. It is suggested that curcumin can effectively improve blood glucose, insulin resistance, and hyperandrogenemia in PCOS [19–21]. Moreover, curcumin reportedly exerts numerous biological effects through multitargeting mechanisms. Among them, targeting ER stress is considered a potential strategy to manage human diseases, including cancer and neurodegenerative and metabolic disorders [22, 23]. However, it is unclear whether curcumin targets ER stress to protect GCs, prevents ER stress, and improves follicular dysfunction in PCOS.

As an effective nonpharmaceutical intervention, aerobic exercise plays an important role in preventing and alleviating many diseases. It improves the body's immune and cardiovascular systems and metabolic functions [24] from both cellular and molecular levels. Physical exercise represents a crucial first step in the treatment of overweight and obese PCOS, and it is not only effective in the treatment of hyperandrogenism and menstrual disturbances but also plays an important role in improving cardiometabolic profile and cardiopulmonary function in PCOS [25, 26]. Long-term exercise training can regulate ER stress and mitochondrial function and prevent apoptosis [27, 28]. Scientific and adequate exercise effectively improves various ER stress-related diseases [29, 30]. Irisin is cleaved from the extracellular domain of the transmembrane receptor fibronectin type III domain-containing 5 (FNDC5) in muscles. Its level increases with exercise and mediates certain favorable effects of physical activity [31]. Irisin is a small polypeptide hormone that shows beneficial effects on energy expenditure and improves metabolic disorders [32, 33]. Recent studies have shown that irisin alleviates ER stress and hepatic fibrosis by inhibiting PERK-mediated HNRNPA1 destabilization [34]. Irisin alleviates pancreatic injury and fibrosis, which is associated with reduced oxidative and ER stress [35]. However, the effect of exogenous irisin on ER stress and the IRE1*α*-XBP1 pathway in ovarian GCs from PCOS-like rats has not yet been elucidated. Therefore, we investigated the effects of oral curcumin administration combined with exercise and the underlying mechanisms as well as determined whether irisin and curcumin could alleviate hyperandrogenism-induced ER stress in PCOS.

## 2. Materials and Methods

### 2.1. Animal Model of PCOS

Specific pathogen-free (SPF) female Sprague Dawley (SD) rats (21 days old) were provided by Shanghai Xipuer-Bikai Laboratory Animal (Shanghai, China). All animal experiments were performed in accordance with the principles and guidelines of the Institutional Animal Care and approved by the Institutional Research Animal Committee of Nanjing University. The rats were maintained in an SPF environment (Jiangsu Key Laboratory of Molecular Medicine) with a 12 : 12 h light/dark cycle at 24°C. Food and water were provided ad libitum. After 3 days of adaptation, all experimental rats were initially divided into control (*n* = 10) (Ctrl. group) and PCOS (*n* = 40) (PCOS group) groups. The control group received 0.2 mL sesame oil subcutaneously (s.c.). PCOS was induced by the administration of 6 mg/100 g body weight dehydroepiandrosterone (DHEA) dissolved in 0.2 mL sesame oil s.c. The daily treatment of both groups lasted for up to 35 consecutive days.

### 2.2. Medicines and Exercise Protocol

All PCOS rats were randomly divided into four groups (*n* = 10) which include the following: PCOS group (no treatment), PCOS+curcumin group (P+C group) (200 mg/kg, intragastric administration; Sigma, Massachusetts, USA), PCOS+exercise group (P+E group) (6 days/week, 1 h/day), and PCOS+curcumin+exercise group (P+C+E group) (4 h of exercise after curcumin intragastric administration); the treatment lasted for 8 weeks.

### 2.3. Estrous Cycle Determination Assays

Vaginal exfoliation cytology was performed on the control and PCOS-like rats during the last 12 days of PCOS induction for 10 consecutive days at approximately 9 am every morning. A sterile, saline-wetted cotton swab was inserted into the rat vagina to approximately 0.5 cm and gently rotated, followed by applying the sample to a glass slide, which was then placed in 95% ethanol for 15 min. After natural air drying, the slide was stained with hematoxylin-eosin (H&E). The estrous cyclicity of the rats was monitored through vaginal smears and assessed under light microscopy.

### 2.4. ELISA Analysis

Fasting blood sugar (FBG) was measured using a glucometer. Then, blood samples were collected, and serum was separated immediately and stored at −80°C for further determination of testosterone (T), LH, FSH, fasting insulin (FINS), and irisin levels using enzyme-linked immunosorbent assay (ELISA) (Elabscience Biotechnology, China). Based on the levels of FINS and FBG, HOMA − IR = INS × FBG/22.5 [36].

### 2.5. Transmission Electron Microscopy (TEM)

The ovarian tissue was fixed in TEM fixative (G1102, Servicebio, China) at 4°C for 4 h. Next, the samples were embedded in agarose, followed by fixation protected from light with 1% OsO_4_ in 0.1 M phosphate buffer solution (PB) (pH 7.4) for 2 h at room temperature. Subsequently, the samples were cut into small clumps of a volume of 1 mm^3^ and dehydrated through a graded (30%-50%-70%-95%-100%-100%) acetone series, after which they were embedded in acetone and EMBed 812 medium (90529-77-4, Pennsylvania, USA). Finally, the sections were placed on copper slot grids and stained with 2% uranyl acetate and lead citrate. Images were visualized by Hitachi HT-7800 transmission electron microscopy (Tokyo, Japan).

### 2.6. Hematoxylin-Eosin (H&E)

Ovarian tissues were fixed, paraffin-embedded, and processed on slides for H&E staining to examine pathological structural alterations of the ovary. Paraffin slices were stained with hematoxylin and eosin in order to examine the pathological structural alterations of the rat ovary under an optical microscope (Leica Microsystems, Germany).

### 2.7. Isolation and Culture of GCs

Female rats (23 days old) were intraperitoneally injected with pregnant mare serum gonadotropin (20 IU), and 48 h after injection, rats were killed by cervical dislocation. Isolation and primary culture methods of GCs were previously described [5, 6]. The primary GC suspensions were cultured in DMEM-F12 medium supplemented with 10% fetal bovine serum (Gibco, Grand Island, USA) and 1% penicillin–streptomycin (Gibco) at 37°C (95% relative humidity, 5% CO_2_).

### 2.8. Cell Viability Assay

GCs were seeded in 96-well plates at a density of 1 × 10^4^ cells/well and then treated with various concentrations of dihydrotestosterone (DHT), curcumin, and irisin (Cayman Chemical, Michigan, USA) for the indicated times. Cell counting kit-8 (CCK-8) solution (10 *μ*L; A311-02-AA, Vazyme Biotech, Nanjing, China) was added to each well, followed by incubation for 4 h at 37°C. Cell viability was then assessed by measuring absorbance at 450 nm using a microplate reader.

### 2.9. Flow Cytometry

Alexa Fluor 488-conjugated Annexin V and propidium iodide (PI) (C1062L, Beyotime, China) were used to assess the apoptosis rates of primary GCs treated with or without DHT or curcumin. The experiments were performed in accordance with the manufacturer's instructions. FITC-Annexin V-positive cells were considered apoptotic cells.

### 2.10. Doses of Drugs and Chemicals

DHT is a more potent androgen than T, and GCs were seeded in 96-well plates at a density of 1 × 10^5^ cells/well and cultured in full culture medium for 48 h. Next, cells were treated with different concentrations of DHT (0.1, 0.5, 1, 2, and 5 *μ*M). According to preexperiments and previous studies [5, 6], it was found that GCs had the best activity when the DHT-induced concentration was 5 *μ*M. Curcumin is a natural polyphenol purchased from Sigma and has a purity of 99%. It is usually soluble in dimethyl sulfoxide (DMSO), organic solvents, or oils. In our vitro study, curcumin powder was dissolved in DMSO and prepared into 50 mM reserve solution and then diluted into 50 *μ*M working solution with cell culture medium. Gradient concentration (5, 10, 15, 20, 25, 30, and 50 *μ*M) was set to detect the cell viability of curcumin on GCs. Finally, the optimal therapeutic concentration was determined.

### 2.11. TUNEL Analysis

TUNEL assays were performed to detect caspase-3 and caspase-12 levels in ovary sections. Fluorescein (FITC) TUNEL Cell Apoptosis Detection Kit (G1501-100T, Servicebio, China) was used according to the manufacturer's instructions. Slides treated with DNase I for 30 min served as positive controls. DAPI was used to stain the nuclei.

### 2.12. Reactive Oxygen Species (ROS) Detection

Generation of ROS was measured based on the converting 2′,7′-dichlorofluorescin diacetate (DCF-DA) to fluorescent 2′,7′-dichlorofluorescin in the presence of peroxides. Cells were plated in 24-well plates. Following treatment with DHT and curcumin, DCF-DA was diluted to 10 *μ*M with serum-free culture medium. The cell culture medium was removed, and 50 *μ*L of diluted DCF-DA was added to each well followed by a 30 min incubation at 37°C. The medium was discarded, and cells were washed with prechilled PBS three times. Images of cells were captured using an Olympus laser scanning confocal microscope (FV3000).

### 2.13. Immunohistochemistry

Ovarian tissues were embedded in paraffin and sectioned at a thickness of 4 *μ*M. After antigen retrieval, dewaxing, and rehydration, sections were treated with 3% hydrogen peroxide (H_2_O_2_) and then 5% BSA. Next, sections were incubated overnight at 4°C with primary antibodies against GRP-78 (1 : 100, WL03157, Wanleibio, Shenyang, China), CHOP (1 : 100, abs135545, Absin, Shanghai, China), IRE1*α* (1 : 200, 27528-1-AP), and p-IRE1*α* (1 : 200, ab124945, Abcam, Cambridge, UK), followed by staining with secondary goat anti-rabbit HRP-labeled IgG. Sections were then stained with diaminobenzidine for 10 min, counterstained with hematoxylin, covered with coverslips, and observed under an optical microscope (Leica Microsystems, Wetzlar, Germany).

### 2.14. Immunofluorescence

Immunofluorescence was conducted as described previously for immunohistochemistry with slight modification. Essentially, after incubation with the primary antibody against CHOP (1 : 200), IRE1*α* (1 : 50), p-IRE1*α*, *Ar* (1 : 200, ab52615, Abcam, Cambridge, UK), *Cyp19α1* (1 : 200, BS6580, Bioworld, Minnesota, USA), and *Cyp11α*1 (1 : 200, BS5680, Bioworld) overnight at 4°C, tissue sections and cells were incubated at 24°C for 2 h with fluorescently labeled secondary antibodies. Nuclei were counterstained with 4′,6-diamidino-2-phenylindole (C1002, Beyotime, Shanghai, China) at a dilution of 1 : 2000 for 30 min. Images were photographed using an Olympus laser scanning confocal microscope (FV3000). Fluorescence intensity was quantified using Image-Pro Plus 6.0 (Media Cybernetics, Maryland, USA).

### 2.15. Western Blotting

Ovarian tissues and GCs were homogenized in RIPA lysis buffer (P0013B, Beyotime) containing 1 mM Pierce™ Phosphatase Inhibitor (B15001, Selleck, Texas, USA) and 0.1% Halt™ Protease Inhibitor Cocktail (B14001, Selleck). The gel electrophoresis system of Bio-Rad (12% SDS polyacrylamide) was used to separate proteins from samples that contained an identical quantity of protein (30 *μ*g); the proteins were then transferred onto polyvinylidenedifluoride membranes (IPVH00010, Merck Millipore, Massachusetts, USA). Target bands were blocked with 5% BSA for 2 h at 24°C. Subsequently, they were incubated with primary antibodies against GRP-78 (1 : 1000), CHOP (1 : 1000), PERK (1 : 1000, 20582-1-AP, Proteintech, Chicago, USA), ATF-6 (1 : 1000, 24169-1-AP), IRE1*α* (1 : 1000), p-IRE1*α* (1 : 1000), XBP1 (1 : 500, WL00708, Wanleibio, Shenyang, China), Bax (1 : 5000, 50599-2-Ig, Proteintech), caspase-9 (1 : 1000, 10380-1-AP), cleaved-caspase-3 (1 : 1000, 66470-2-Ig), *Ar* (1 : 1000), *Cyp19α1* (1 : 1000), *Cyp11α1* (1 : 1000), and GAPDH (1 : 5000, MB9231, Bioworld) overnight at 4°C, followed by the addition of HRP-labeled secondary antibodies. Blots were visualized using chemiluminescent detection. Densitometric analysis was performed using Image J.

### 2.16. Reverse Transcription Quantitative Polymerase Chain Reaction (RT-qPCR)

Total RNA from tissues and cells was extracted using TRIzol reagent (R401-01, Vazyme Biotech), and cDNA was synthesized using a reverse transcription kit (R223-01, Vazyme Biotech). RT-qPCR was performed using the SYBR Green PCR Master Mix (Q441-02, Vazyme Biotech), and the primers are shown in (Table 1). The *ΔΔ*CT method was used to quantify gene expression, with the threshold cycle denoted by CT. The expression of the target genes was normalized to that of control genes with the lowest CT and reported as 2^−*ΔΔ*CT^. *GAPDH* was used as an internal control.

### 2.17. Statistics

Data are expressed as the mean ± SEM. Results from different groups were compared using analysis of variance with Bonferroni adjustments, in which *P* < 0.05 was regarded as statistically significant.

## 3. Results

### 3.1. Treatment with Curcumin and Aerobic Exercise Improve Ovarian Morphology and Serum Hormone Levels in PCOS Rats

Cytological examination showed that compared with the DHEA-induced PCOS-like rats, the estrus cycle of the control rats was normal, presenting a regular proestrus-estrus-metestrus-diestrus cycle. In contrast, DHEA-induced PCOS-like rats were completely acyclic, and most of them were in estrus (Sup-Fig. S1A and S1B). Next, we characterized ovarian histology using H&E staining. Control rats showed synchronized follicular development and abundant corpora lutea and normal follicular maturation and ovulation. However, PCOS-like rat ovaries had more atretic follicles and a thinned follicular granular layer, almost without corpus luteum compared with the control group (Sup-Fig. S1C). Additionally, DHEA-induced PCOS-like rats exhibited disordered serum hormone levels according to ELISA. Serum T and LH levels were markedly elevated, and serum FSH levels were decreased (Sup-Fig. S1D and S1E).

**Figure 1:**
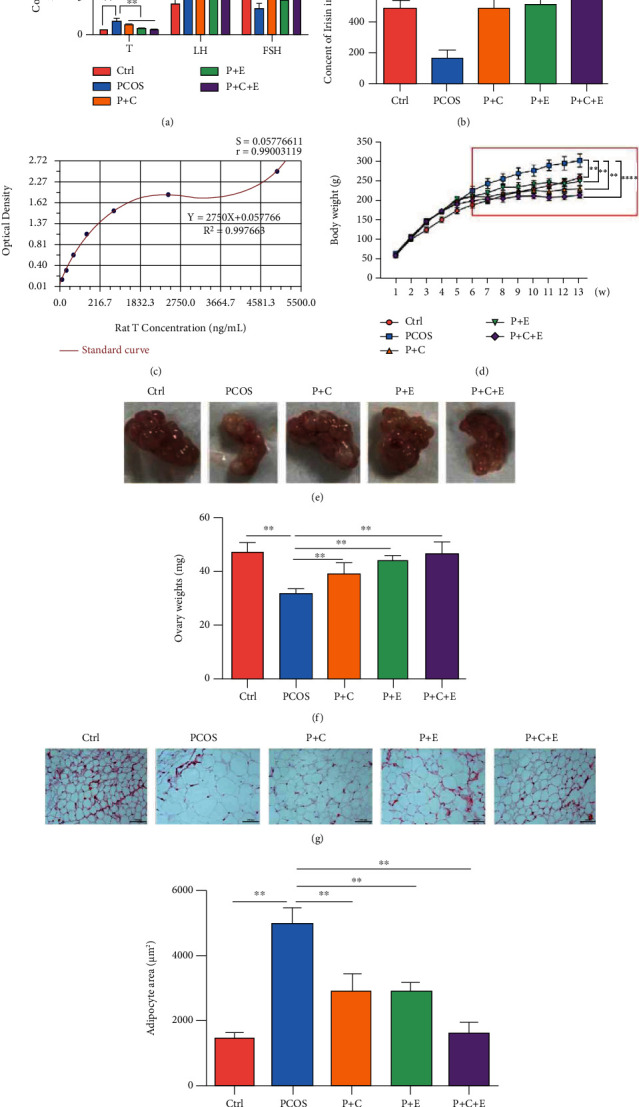
Curcumin and aerobic exercise improve serum hormone levels and ovarian morphology in PCOS-like rat. PCOS-like rats received 8 weeks of curcumin gavage, treadmill exercise, and curcumin combined with treadmill exercise treatment. (a) Serum T, FSH, and LH levels were analyzed using enzyme-linked immunosorbent assay kits. (b) The level of serum irisin immediately after curcumin gavage and treadmill exercise by an enzyme-linked immunosorbent assay analysis. (c) Quantification of enzyme-linked immunosorbent assay of serum irisin in all experimental group rats. (d) Rat body weights were measured in all experimental groups after 8 weeks of treatment. (e) Photographs of the morphology of the ovaries and from each treatment group were shown. (f) Average weight of both ovaries was measured. (g, h) Abdominal fat in all experimental group rats was assessed by H&E staining (20x) after 8 weeks and the quantitative analysis. Three independent experiments were performed with similar results. Data are shown as mean ± SEM. ^∗∗^*P* < 0.05 and ^∗∗∗∗^*P* < 0.01.

After 8 weeks of curcumin and exercise treatment, we measured serum T, LH, FSH, and FINS levels using ELISA. Serum T and LH levels were markedly decreased, and serum FSH levels were elevated in the treatment group (Figure 1(a)). Furthermore, compared with the control group, the FBG, FINS, and HOMA-IR in the PCOS group were increased. Relative to the PCOS group, the P+C group, P+E group, and P+C+E group had decreased FBG, FINS, and HOMA-IR levels (Table 2). Irisin is secreted by muscle cells; its levels increase with exercise, and it mediates certain favorable effects of physical activity [28]. Therefore, we measured serum irisin levels immediately after treadmill exercise using ELISA. Serum irisin levels in PCOS-like rats increased significantly after exercise treatment compared with those in PCOS-like rats. Interestingly, serum irisin also increased after curcumin treatment (Figures 1(b) and 1(c)). Additionally, DHEA-induced PCOS-like rats treated with curcumin and exercise had lower body weights than untreated PCOS-like rats (Figure 1(d)). Macroscopic view of ovary was shown that PCOS-like rat ovaries were dramatically smaller compared to the control group, which was consistent with our previous results [5]. However, as expected, the ovarian weight and morphology were markedly improved after treatment with curcumin and exercise (Figures 1(e) and 1(f)). Further, after the PCOS-like rats received curcumin and exercise treatment, lipid droplets in abdominal fat became smaller with a normal size using H&E staining (Figures 1(g) and 1(h)**).**

### 3.2. ER Stress Is Activated in the Ovaries of DHEA-Induced PCOS Rats and May Cause Ovarian Dysfunction

**Figure 2:**
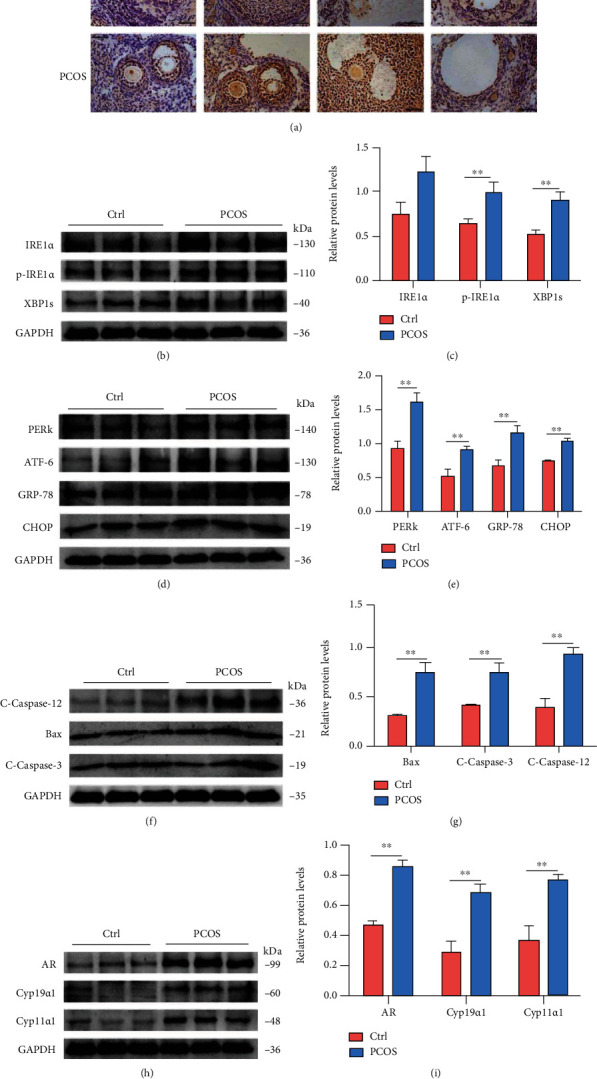
ER stress is activated in ovaries of DHEA-induced PCOS rats and may cause ovarian dysfunction. (a) The levels of IRE1*α*, p-IRE1*α*, GRP-78, and CHOP were measured with immunohistochemical staining. (b–e) The expression of IRE1*α*, p-IRE1*α*, XBP1, PERK, ATF-6, GRP-78, and CHOP in ovaries was assessed by western blot assay. (f, g) The expression of apoptosis proteins cleaved-caspase-12, Bax, and cleaved-caspase-3 in ovaries was assessed by western blot assay. (h, i) The expression of *Ar*, *Cyp11α1*, and *Cyp19α1* in ovaries was assessed by western blot assay. Three independent experiments were performed with similar results. Data are shown as mean ± SEM. ^∗∗^*P* < 0.05.

After the PCOS-like rat model was successfully established, we determined whether there was excessive ER stress in the ovaries. First, we undertook to measure IRE1*α*, p-IRE1*α*, GRP-78, and CHOP expression in the ovaries of the two groups using immunohistochemical staining (Figure 2(a)). Simultaneously, we measured IRE1*α*, p-IRE1*α*, XBP1, PERK, ATF-6, GRP-78, and CHOP expression levels using western blotting, which were all upregulated compared to control levels (Figures 2(b) and 2(e)**)**. The expression of the apoptosis-associated proteins Bax and cleaved-caspase-3 was also increased in PCOS rats (Figures 2(f) and 2(g)). Furthermore, in the PCOS group, the expression of the GC-related genes, including *Ar*, *Cyp11α1*, and *Cyp19α1*, was markedly increased compared to that in the control group (Figures 2(h) and 2(i)**)**.

### 3.3. PCOS Rat Ovarian Dysfunction Is Improved via a Combined Curcumin and Exercise Treatment Possibly via Suppressing Excessive ER Stress

**Figure 3:**
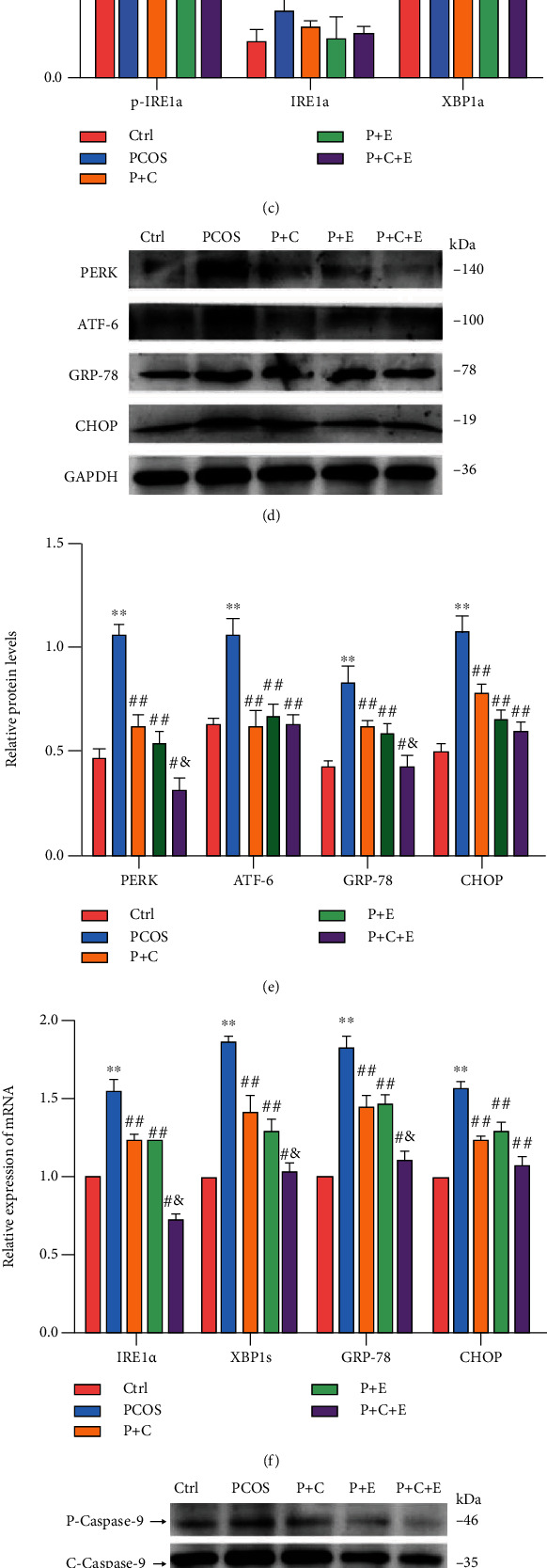
PCOS-like rat ovarian dysfunction is improved via a combined curcumin and exercise treatment possibly via excessive ER stress suppression. PCOS-like rats received 8 weeks of curcumin gavage, treadmill exercise, and curcumin combined with treadmill exercise treatment. (a) Transmission electron microscopic analysis of follicles from each group (×3.0 k and ×8.0 k). (b–f) The expression of IRE1*α*, p-IRE1*α*, XBP1, PERK, ATF-6, GRP-78, and CHOP in ovaries was assessed by western blot and qRT-PCR assay. (g, h) The levels of Bax, cleaved-caspase-3, and caspase-9 by western blot. Three independent experiments were performed with similar results. Data are shown as mean ± SEM. ^∗∗^*P* < 0.05 vs. Ctrl. ^##^*P* < 0.05 vs. PCOS. ^#&^*P* < 0.05 vs. P+C/P+E.

**Figure 4:**
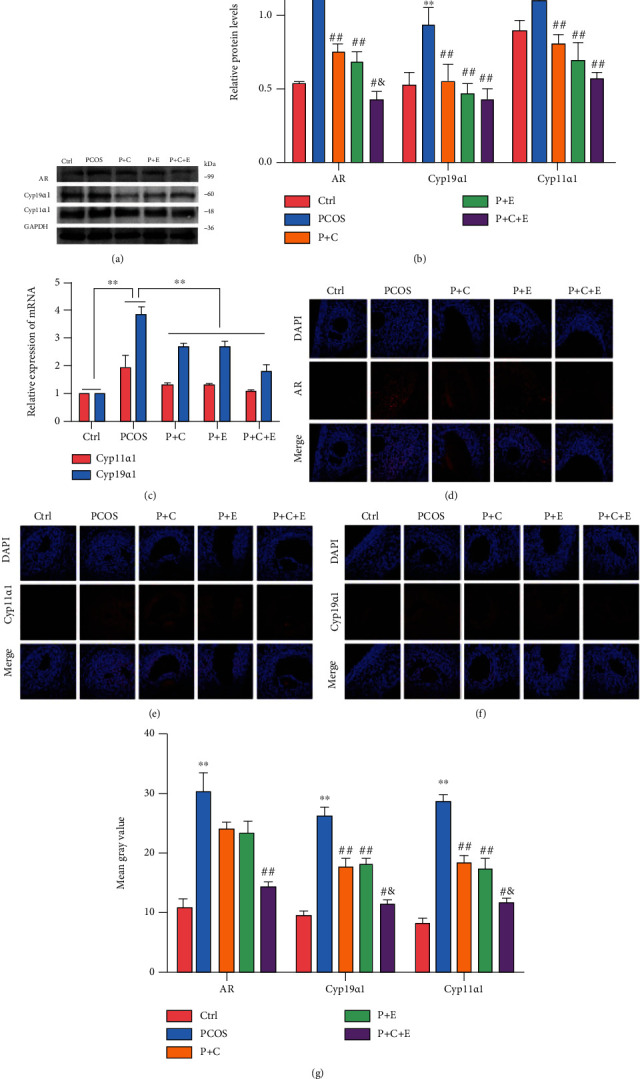
PCOS-like rat ovarian dysfunction was improved by treatment with curcumin combined with exercise possibly via the suppression of ER stress. PCOS rats received 8 weeks of curcumin gavage, treadmill exercise, and curcumin combined with treadmill exercise treatment. (a–g) The expression of *Ar*, *Cyp11α1*, and *Cyp19α1* in ovaries was assessed by western blot assay, qRT-PCR analysis, and immunofluorescence. Three independent experiments were performed with similar results. Data are shown as mean ± SEM. ^∗∗^*P* < 0.05. ^∗∗^*P* < 0.05 vs. Ctrl. ^##^*P* < 0.05 vs. PCOS. ^#&^*P* < 0.05 vs. P+C/P+E.

There were significantly reduced numbers of atretic follicles in PCOS-like rat ovaries after curcumin and exercise treatment using H&E staining, whereas the corpus luteum was increased, and the granular layer of the follicle thickened (Sup-Fig. S2A). Next, we observed changes in the ovary ultrastructure using TEM. The control group displayed normal mitochondria, with an organized appearance of normal mitochondrial cristae and many parallel membrane layers. However, in the PCOS group, with mitochondrial abnormalities (lack of definite crest structure) and expansion of the ER, curcumin and exercise treatments effectively reduced this stress and significantly prevented mitochondrial and ER damage (m: mitochondrion; s: smooth endoplasmic reticulum; and r: rough endoplasmic reticulum) (Figure 3(a)). p-IRE1*α* and GRP-78 levels were significantly decreased in the ovary from mice treated with curcumin and exercise (Sup-Fig. S2B). Moreover, expression of ER stress-associated markers in ovarian tissues was measured using western blotting and RT-qPCR. IRE1*α*, p-IRE1*α*, PERK, ATF-6, GRP-78, and CHOP were all significantly decreased at both protein and mRNA levels in rats treated with curcumin and exercise (Figures 3(b)–3(f)). Next, we measured the levels of apoptosis-related markers. Bax, cleaved-caspase-3, and caspase-9 were all significantly decreased at both protein and mRNA levels in rats treated with curcumin and exercise (Figures 3(g) and 3(h)). We further performed TUNEL analysis in ovarian sections. Compared with the control group, TUNEL-positive cells in ovarian tissues of PCOS group were significantly increased, but after curcumin or exercise alone, there was no change in TUNEL-positive cells, and the TUNEL-positive cells were decreased in curcumin and exercise combined treatment group. In addition, the expression levels of cleaved-caspase-3 and caspase-12 were significantly increased in the model group but did not decrease significantly after curcumin and exercise treatment (Sup-Fig. S2C-S2D). The expression of GC-related genes, including *Ar*, *Cyp11α1*, and *Cyp19α1*, was markedly increased in the ovarian tissue of PCOS-like rat and was decreased after curcumin and exercise treatment, as confirmed using western blotting and RT-qPCR (Figures 4(a)–4(c)). Then, we detected the expression of these genes in the ovarian tissues of each group by immunofluorescence. Compared with the control group, the expression levels of *Ar*, *Cyp11α1*, and *Cyp19α1* in the ovarian tissues of rats in the model group were significantly increased. Relative to the rats in model group, the expression levels of *Ar*, *Cyp11α1*, and *Cyp19α1* were not significantly different in P+C and P+E groups but significantly decreased in the P+C+E group (Figures 4(d)–4(g)).

### 3.4. DHT Induces Apoptosis Mediated by Excessive ER Stress, Resulting in GC Dysfunction

Our previous research showed that hyperandrogenism can induce ovarian GC pyroptosis [6]. We treated GCs with various DHT concentrations (0.1, 0.5, 1, 2, and 5 *μ*M) for 48 h. As expected, GC cell viability was dose-dependently decreased by DHT, which induced the highest rate of cell death at 5 *μ*M (Sup-Fig. S3A). The apoptosis rate of DHT-treated GCs was also analyzed by PI and FITC-Annexin V staining followed by microscopic examination (Sup-Fig. S3B). The expression of the apoptotic proteins Bax and cleaved-caspase-3 was markedly enhanced in 0.5 or 5 *μ*M DHT-treated GCs (Sup-Fig. S3C, S3D). To further confirm excessive ER stress, we examined IRE1*α*, p-IRE1*α*, XBP1, PERK, ATF-6, GRP-78, and CHOP expression at both the protein and mRNA levels in GCs treated with 5 *μ*M DHT using western blotting and RT-qPCR (Sup-Fig. S3E-S3G). High-level DHT induced excessive ER stress in ovarian GCs, leading to GC apoptosis, and dysregulated the steroid synthases *Cyp11α1* and *Cyp19α1* (Sup-Fig. S3H, S3I).

### 3.5. Curcumin Alleviates Tunicamycin-Induced Excessive ER Stress in GCs, Thereby Reducing Apoptosis

Tunicamycin (TM) is commonly used to create ER stress models [37]. We used an in vitro system to investigate ER stress-mediated apoptosis in rat ovarian GCs induced by TM, as a positive control, at various concentrations (0.5, 1, 2, 5, and 10 *μ*g/mL) for 24 h [38]. TM induced the highest rate of GC death at 2 *μ*g/mL (Sup-Fig. S4A). Furthermore, treatment with TM in GCs increased ROS levels as determined by the DCF-DA probe (Sup-Fig. S4B, S4C). High IRE1*α*, p-IRE1*α*, GRP-78 and CHOP levels were also observed in GCs after TM treatment (Sup-Fig. S4D, S4E). Further, the expression of the apoptotic protein Bax in GCs was significantly upregulated (Sup-Fig. S4F, S4G). Next, we treated GCs with various curcumin concentrations (5, 10, 15, 20, 25, 30, and 50 *μ*M) in the presence of TM for 48 h. First, GC viability after curcumin treatment was analyzed using CCK-8, and its optimal induction concentration (curcumin: 20 *μ*M) was determined (Sup-Fig. 4H). Curcumin treatment reduced TM-induced apoptosis at concentrations of 5-20 *μ*M and decreased protein expression levels of Bax in GCs. Importantly, curcumin inhibited GRP-78 expression (Sup-Fig. S4I, S4J).

### 3.6. Curcumin Alleviates DHT-Induced ER Stress, Apoptosis, and Dysfunction in GCs

**Figure 5:**
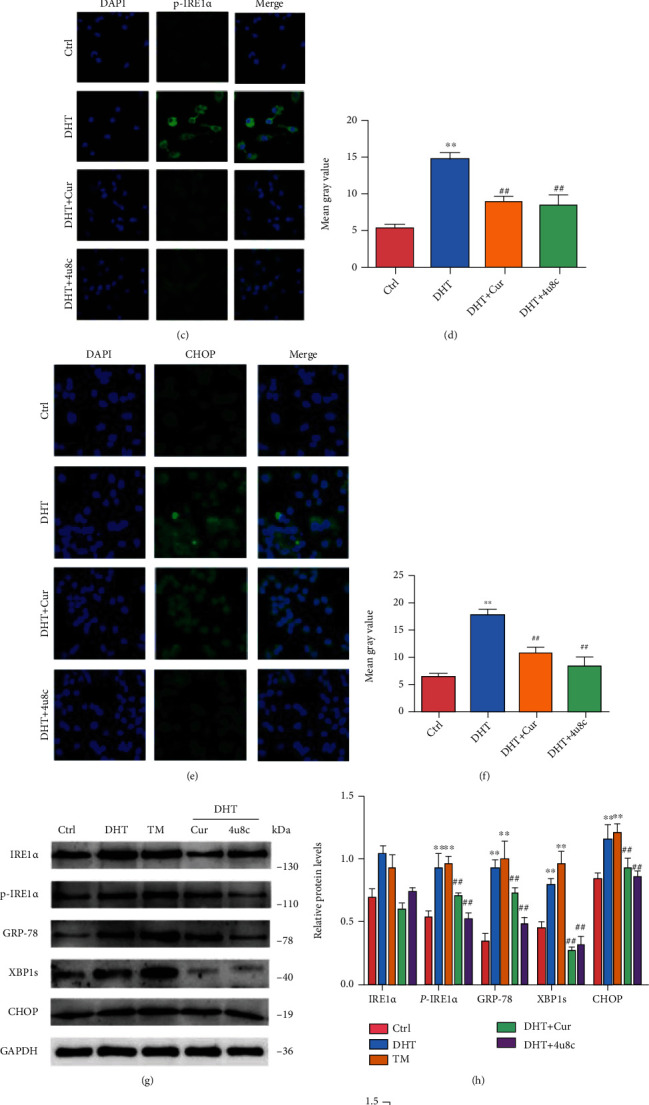
Curcumin alleviates DHT-induced ER stress and apoptosis. DHT-induced GCs were treated with different concentrations of curcumin or 4*μ*8C. (a) Cell viability of granulosa cells after curcumin and irisin treatment was analyzed by CCK-8 kits. (b) Representative flow cytometry scatter plots of propidium iodide (PI) (*Y* axis) vs. Annexin V-fluorescein isothiocyanate (FITC) (*X* axis). (c–f) Levels of p-IRE1*α* and CHOP in GCs with curcumin treatment were measured by immunofluorescence staining (60x). (g, h) The expression of IRE1*α*, p-IRE1*α*, XBP1, GRP-78, and CHOP was assessed by western blot assay. (i, j) The expression of cleaved-caspase-12, Bax, and cleaved-caspase-3 was assessed by western blot assay. Three independent experiments were performed with similar results. Data are shown as mean ± SEM. ^∗∗^*P* < 0.05. ^∗∗^*P* < 0.05 vs. Ctrl. ^##^*P* < 0.05 vs. DHT/TM.

DHT induces GC apoptosis mediated by excessive ER stress, resulting in GC dysfunction (Sup-Fig. 3). To investigate the protective effects of curcumin against DHT-induced damage, GCs were cotreated with curcumin (5, 10, 15, and 20 *μ*M) and DHT (5 *μ*M) for 48 h. GC viability was approximately reduced by 40% after incubation with 5 *μ*M DHT for 24 h [6]. Cell viability was rescued after curcumin treatment (Figure 5(a)). To further confirm the protective role of curcumin is dependent on the activation of the IRE1*α*-XBP1 pathway, we used specific IRE1*α*-XBP1 inhibitor 4*μ*8C (10 *μ*M, HY-19707, Shanghai, China) in vitro studies. GCs were treated with 20 *μ*M curcumin or 10 *μ*M 4*μ*8C in the presence of 5 *μ*M DHT for 24 h to determine whether it could reduce excessive ER stress, thereby reducing apoptosis. Both Annexin V and PI were significantly decreased in curcumin- or 4*μ*8C-treated GCs (Figure 5(b)). Furthermore, p-IRE1*α* and CHOP levels were examined using immunofluorescence assays (Figures 5(c)–5(f)). The IRE1*α*, p-IRE1*α*, GRP-78, and CHOP protein levels were also assessed using western blotting. As expected, curcumin significantly reduced the ER stress induced by DHT in GCs (Figures 5(g) and 5(h)). Furthermore, caspase-12, Bax, and cleaved-caspase-3 levels were markedly decreased in GCs after curcumin treatment (Figures 5(i) and 5(j)). In addition, the expression of *Cyp11α1* and *Cyp19α1* was examined by immunofluorescence assays. The expression of these genes was markedly decreased in GCs by treatment with curcumin (Sup-Fig. S5A, S5D).

We have previous shown that DHT-induced GCs are subjected to OS and mitochondrial dysfunction [5]. Next, we assessed ROS levels in GCs using the DCF-DA probe. H_2_O_2_ is commonly used in models of OS-induced apoptosis. Here, we treated GCs with 500 *μ*M H_2_O_2_ as a positive control to induce OS [38, 39]. The DHT-induced increase in ROS was consistent with that obtained upon H_2_O_2_ treatment. Further, curcumin (20 *μ*M) treatment reduced ROS levels (Sup-Fig. 5E, 5F). This result suggested decreased ROS levels in GCs treated with curcumin. Mitochondrial membrane potential dysfunction, measured using JC-1 monomers, was induced by treatment with H_2_O_2_ or DHT. In contrast, mitochondrial membrane potential remained intact in blank control and curcumin/4*μ*8C treatment groups (Sup-Fig. S5G-S5H). These results suggest that curcumin reduces ER stress, OS, and mitochondrial dysfunction in DHT-induced GCs, thereby improving ovarian GCs dysfunction.

### 3.7. Irisin Possibly Alleviates GC Apoptosis by Downregulating the IRE1*α*-XBP1 Pathway

**Figure 6:**
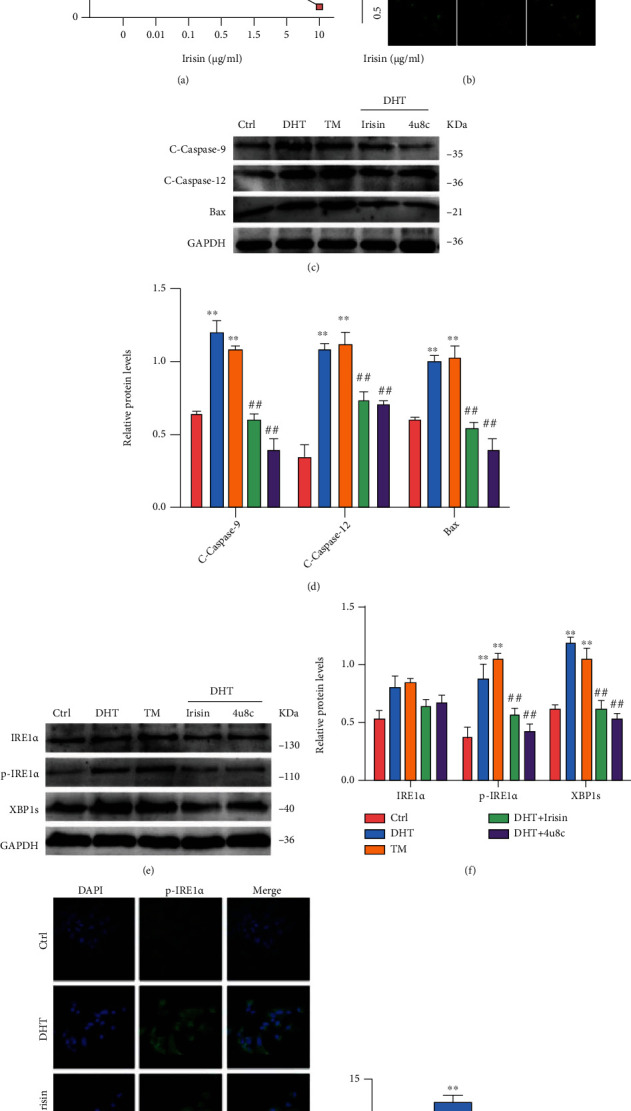
Irisin possibly alleviates GC apoptosis by downregulating the IRE1*α*-XBP1 pathway. DHT-induced GCs were treated with different concentrations of irisin. (a) Cell viability of GCs after irisin treatment was analyzed by CCK-8 kits. (b) GCs were incubated with Annexin V-FITC and PI. The cells were imaged for apoptosis detection using a FV3000 Olympus microscope. (c, d) The expression of caspase-9, caspase-12, and Bax was assessed by western blot assay. (e, f) The expression of IRE1*α*, p-IRE1*α*, and XBP1 was analyzed by western blot assay. (g, h) The expression of p-IRE1*α* was analyzed by immunofluorescence staining (60x). Three independent experiments were performed with similar results. Data are shown as mean ± SEM. ^∗∗^*P* < 0.05 vs. Ctrl. ^##^*P* < 0.05 vs. DHT/TM.

Our data showed that serum irisin in PCOS-like rats increased significantly after exercise (Sup-Fig. S1B). Thus, GCs were treated with various irisin concentrations (0.01, 0.1, and 0.5 *μ*g/mL), and GC viability after irisin treatment was analyzed using CCK-8 (Figure 6(a)). Irisin treatment reversed DHT-induced apoptosis of GCs (Figure 6(b)). Thus, GCs were treated with 0.5 *μ*g/mL irisin or 10 *μ*M 4*μ*8C in the presence of 5 *μ*M DHT for 24 h. As expected, irisin treatment exerted an antiapoptotic effect on DHT-induced GCs, and Bax, caspase-9, and caspase-12 levels were decreased (Figures 6(c) and 6(d)). To further determine whether irisin reduces GC apoptosis by downregulating IRE1*α*-XBP1 pathway proteins after ER stress, we measured IRE1*α*, p-IRE1*α*, and XBP1 protein levels in GCs treated with irisin using western blotting (Figures 6(e) and 6(f)). Moreover, p-IRE1*α* level was examined using immunofluorescence assay (Figures 6(g) and 6(h)). As expected, irisin significantly reduced IRE1*α*-XBP1 induced by DHT in GCs. These results suggest that irisin alleviates GC apoptosis by alleviating ER stress and downregulating the IRE1*α*-XBP1 pathway.

## 4. Discussion

ER homeostasis is an important condition during oocyte development. However, during oocyte maturation, many factors affect ER homeostasis, leading to ER stress. Appropriate ER stress is conducive to the growth and development of follicles, the corpus luteum, and embryo, whereas excessive ER stress leads to follicular atresia, luteal dysfunction, embryo development disadvantages, and implantation failure, even leading to infertility and other ovarian diseases. Ovarian dysfunction in PCOS is related to ER stress [10], but the molecular mechanisms have not been clarified. A report showed that the IRE1 (IRE1, XBP1, and JNK) pathway is involved in autophagy and damage of mouse ovaries induced by microcystin-LR [40]. Previous studies have shown that testosterone induces ER stress and unfolded protein response (UPR) activation [12]. Our findings show that PCOS follicular development disorders might be caused by long-term, hyperandrogen-induced ER stress in GCs, which might cause apoptosis. Further, genes encoding proteins related to follicular development (*Ar*, *Cyp11α1*, and *Cyp19α1*) were abnormally expressed. Moreover, curcumin and aerobic exercise could relieve ER stress, reduce GCs apoptosis, and improve ovarian function.

It is interesting to explore the mechanism underlying the combination of drugs and exercise, speculating that a combination of both might alleviate ER stress in different diseases. Exercise or rutin different organs and tissues alone do not change the expression of IRE1*α* in the liver of high-fat diet- (HFD-) fed mice, but their combination significantly reduces its expression [41]. Exercise combined with resveratrol regulates ER stress via different pathways in an exercise intensity-dependent manner in a nonalcoholic fatty liver disease model induced by HFD [42]. Additionally, curcumin supplementation in humans is likely safe and beneficial for sports and physical activity [43]. However, the oral bioavailability of curcumin is reported to be low in rats [19]. Therefore, curcumin was administered orally at 200 mg/kg/day in this study. This is equivalent to a medium-high-level dose and was determined based on preliminary experiments and references [44]. There are some reports indicating that the oral bioavailability of curcumin can be improved using solid lipid nanoparticles [45]. This provides a method to improve the utilization rate of curcumin for its application in functional foods and medicines. ROS generation can trigger apoptotic cell death by activating the ER stress pathway. Curcumin (20 *μ*M) treatment reduced hyperandrogen-induced ROS levels in GCs. The fact that ROS-mediated pathways play a critical role in apoptosis induced by curcumin is consistent with a previous report [46]. Moreover, curcumin significantly reduced GRP-78 and CHOP at both protein and mRNA levels induced by DHT in GC, and the expression of the apoptotic proteins Bax, cleaved-caspase-3, and caspase-9 was markedly decreased. In addition, DHT reportedly induces OS and mitochondrial dysfunction in GCs [5]. We found that curcumin could not only relieve ER stress of GCs but also reduce GC OS levels and increase the intracellular mitochondrial membrane potential. Therefore, curcumin, as an antioxidant and modulator of ER stress, might be a good candidate for PCOS treatment.

Presently, there is no clear conclusion regarding the impact of different exercise methods, exercise intensity, and exercise duration on ER stress intensity and stress duration in different tissues. In an 8-week swimming training study on HFD-fed rats, aerobic exercise is found to significantly reduce ER stress in rat liver tissue and testicular fat [47]. Three months of aerobic exercise is found to reduce GRP-78, IRE1, and p-eIF2*α* mRNA and protein levels in subcutaneous fat tissue and blood mononuclear cells of obese adults [48]. Treadmill exercise training can reduce GRP-78, p-PERK, p-eIF2*α*, ATF-6, XBP1, CHOP, and cleaved-caspase-3 expression to improve the cardiovascular function and reduce myocardial infarction in rats [49]. Correspondingly, 3 weeks of high-intensity treadmill exercise can strengthen the ER stress degree in the brain tissue of obese mice fed with a HFD, with the hypothalamus presenting the highest ER stress degree [50]. In our study, PCOS rats underwent 8 weeks of treadmill exercise, running at a speed of 20 m/min for 6 days/week and exercising for 60 min/day. After 8 weeks of aerobic exercise, macroscopically, rats showed decreased body weight, ovarian weight, and abdominal fat. The polycystic morphology of ovaries also improved. Additionally, aerobic exercise significantly improved excessive ER stress in the ovaries of PCOS-like rats. However, in the absence of data on the exercise type, intensity, or duration which yields benefits in women with PCOS, recommendations for exercise are vague and are therefore challenging to implement. Well-designed trials are required to guide clinical management in women of reproductive age with PCOS. Focus should be on the role of exercise therapy in the prevention of PCOS to help develop personalized and appropriate types and intensities of exercise and targeted exercise prescriptions for disease prevention.

Irisin might play an important role in the occurrence and development of PCOS. Our data show that irisin treatment reduced GC apoptosis by downregulating the IRE1*α*-XBP1 pathway after ER stress. However, factors secreted by muscles also include insulin-like growth factor-I, fibroblast growth factor-2, myostatin, and interleukin-6, among others [51, 52]. Whether these factors produced upon exercise might also have a certain correlation with the occurrence and development of PCOS is unknown. In our study, it is worth noting that an increase in serum irisin levels was not only caused by exercise but also by curcumin gavage. This implies the potential therapeutic applications of curcumin for the treatment of PCOS or other energy metabolism diseases. To date, whether curcumin can increase serum irisin levels in PCOS rats has not been reported. The specific mechanism requires further discussion. The ER is extremely sensitive to microchanges in the internal and external environment of cells. ER stress is immediately activated when cell homeostasis is unbalanced and regulates the internal environment by activating the UPR signaling pathway.

In this study, our data confirm that hyperandrogen induced ovarian GC apoptosis in PCOS-like rats, which was mediated by excessive ER stress and IRE1*α*-XBP1 pathway activation, resulting in follicle development disorders. Both curcumin gavage and aerobic exercise improved ovarian function via inhibiting the hyperandrogen-activated ER stress IRE1*α*-XBP1 pathway. DHT-induced ER stress was mitigated by curcumin/irisin or 4*μ*8C treatments in primary GCs, in which upregulation of follicular development-related genes, including *Ar*, *Cyp11α1*, and *Cyp19α1*, was suppressed. In summary, curcumin and aerobic exercise (irisin) can alleviate hyperandrogenism-induced ER stress and suppress the IRE1*α*-XBP1 pathway, which prevented ovarian GC apoptosis in PCOS-like rats, leading to the improvement in the ovarian microenvironment and promotion of follicular development.

## Figures and Tables

**Table 1:** Sequences of primers designed for RT-qPCR.

Genes	Forward	Reverse
*PERK*	5′-GCCGACGATCAAATGGAAGC-3′	5′-GTGGGGCTGAGGATGGAAAA-3′
*ATF-6*	5′-TCAGCTGATGGCTGTCCAGT-3′	5′-TGATGTGGAGGATCCTGGTG-3′
*IRE1α*	5′-AACACACCGACCACCGTATC-3′	5′-AGGGTACTGGGTAAGGTCTC-3′
*XBP1s*	5′-TAGAAAGAAAGCCCGGATGA-3′	5′-TCTCAATCACAAGCCCATGA-3′
*GRP-78*	5′-GCCAACTGTAACAATCAA-3′	5′-GCTGTCACTCGGAGAATA-3′
*CHOP*	5′-GCTGGAAGCCTGGTATG-3′	5′-CTTTGGGATGTGCGTGT-3′
*Cyp11α1*	5′-GGATGCGTCGATACTCTTCTCA-3′	5′-GGACGATTCGGTCTTTCTTCCA-3′
*Cyp19α1*	5′-AACCCGAGCCTTTGGAGAA-3′	5′-GGCCCGTCAGAGCTTTCA-3′
*GAPDH*	5′-ACTCACTCTTCTACCTTTGATGCT-3′	5′-TGTTGCTGTAGCCAAATTCA-3′

**Table 2:** The levels of FBG, FINS, and HOMA-IR in all experimental groups.

Group	FBG	FINS	HOMA-IR
Ctrl	3.76 ± 0.47	20.83 ± 2.03	3.49 ± 0.58
PCOS	4.68 ± 1.20	36.13 ± 6.06	7.56 ± 2.13
P+C	4.02 ± 0.97	23.07 ± 2.68	4.09 ± 0.88
P+E	4.04 ± 0.62	21.72 ± 4.77	3.97 ± 1.33
P+C+E	3.86 ± 0.76	20.52 ± 2.10	3.55 ± 0.97

## Data Availability

The datasets generated and/or analyzed during the current study are available from the corresponding author on reasonable request. In addition, all data generated or analyzed during this study are included in this published article.

## Supplementary Materials

Supplementary Materials Supplementary data are available at Oxidative Medicine and Cellular Longevity online. Supplementary Figure 1: DHEA-induced PCOS-like rats have irregular estrous cycles, polycystic ovaries, and disordered hormone levels. (A) The cell types in vaginal smears corresponding to each period. a: proestrus, predominantly consisting of nucleated epithelial cells; b: estrus, with cornfield squamous epithelial cells; c: metestrus, consisting of cornified squamous epithelial cells and predominance of leukocytes; d: diestrus, consisting predominantly of leucocytes (as shown by the arrow). (B) Estrus cycle was examined in control and PCOS groups of mice by evaluating the cell types in vaginal smears. (C) Ovarian and follicular morphology was assessed by H&E staining (2.5x, 5x, and 10x). (D) Linear regression equation shows the relationship between the O.D. values at the wavelength of 450 nm and the concentrations of rat serum T, FSH, and LH. (E) Quantification of enzyme-linked immunosorbent assay of serum in the two groups rats. Three independent experiments were performed with similar results. Data are shown as mean ± SEM. ^∗∗^*P* < 0.05. Supplementary Figure 2: PCOS-like rat ovarian dysfunction is improved via a combined curcumin and exercise treatment. (A) Ovarian and follicular morphology was assessed by H&E staining (5x and 10x). (B) The levels of GRP-78 and p-IRE1*α* were measured with immunohistochemical staining. (C, D) TUNEL analysis of the levels of caspase-12 and cleaved-caspase-3 on ovarian sections. Three independent experiments were performed with similar results. Supplementary Figure 3: DHT induces granulosa cell apoptosis mediated by excessive ER stress resulting in GC dysfunction. (A) Cell viability of granulosa cells after tunicamycin-induced was analyzed by CCK-8 kits. (B) GCs were incubated with Annexin V-FITC and PI. The cells were imaged for apoptosis detection using a FV3000 Olympus microscope. (C, D) The apoptosis factors Bax and cleaved-caspase-3 significantly increased by western blot assay. (E, F) The expression of steroid synthase *Cyp11α1* and *Cyp19α1* by western blot assay. (G–I) The protein and mRNA levels of CHOP, GRP-78, ATF-6, PERK, IRE1*α*, p-IRE1*α*, XBP1, and caspase-12 were assessed by western blot assay and qRT-PCR assay. Three independent experiments were performed with similar results. Data are shown as mean ± SEM. ^∗∗^*P* < 0.05 and ^∗∗∗∗^*P* < 0.01. Supplementary Figure 4: curcumin alleviates tunicamycin- (TM-) induced excessive ER stress in GCs, thereby reducing apoptosis. (A) Cell viability of granulosa cells after tunicamycin-induced was analyzed by CCK-8 kits. (B, C) ROS generation in GCs following tunicamycin treatment was measured using the DCF-DA probe. DCF-DA fluorescence (green fluorescence) was measured by confocal microscopy (20x). Images are representative of three independent experiments with similar results. Quantification of the fluorescence is shown. (D, E) The expression of GRP-78, CHOP, IRE1*α*, and p-IRE1*α* in GCs by different concentrations of tunicamycin treatment by western blot assay. (F, G) The expression level of apoptosis protein Bax was detected by western blot assay. GCs were treated with curcumin in the presence of TM at 0.2 *μ*g/mL. (H) Cell viability of GCs after curcumin treatment was analyzed by CCK-8 kits. (I, J) The protein level of GRP-78 and Bax in GCs after curcumin treatment was analyzed by western blot. (K, L) Three independent experiments were performed with similar results. Data are shown as mean ± SEM. ^∗∗^*P* < 0.05 and ^∗∗∗∗^*P* < 0.01. Supplementary Figure 5: curcumin alleviates DHT-induced ROS and MMP and improved ovarian function. (A–D) The expression levels of *Cyp11α1* and *Cyp19α1* in GCs with curcumin or 4*μ*8C treatment were measured by immunofluorescence staining (60x). (E, F) ROS generation in granulosa cells following tunicamycin treatment was measured using the DCF-DA probe. DCF-DA fluorescence (green fluorescence) was measured by confocal microscopy (20x). Images are representative of three independent experiments with similar results. (G, H) Mitochondrial membrane potential was analyzed by the ratio of JC-1 monomers/polymers (60x). Three independent experiments were performed with similar results. Data are shown as mean ± SEM. ^∗∗^*P*  < 0.05. ^∗∗^*P* < 0.05 vs. Ctrl. ^##^*P* < 0.05 vs. DHT/H_2_O_2_.
