# Supplementary material for: Curcumin in Combination with Aerobic Exercise Improves Follicular Dysfunction via Inhibition of the Hyperandrogen-Induced IRE1α/XBP1 Endoplasmic Reticulum Stress Pathway in PCOS-Like Rats
Source: Oxid Med Cell Longev. 2021 Dec 26;2021:7382900. doi: 10.1155/2021/7382900 (PMC8720591; doi:10.1155/2021/7382900)
Supplement: Supplementary Materials — Supplementary data are available at Oxidative Medicine and Cellular Longevity online. Supplementary Figure 1: DHEA-induced PCOS-like rats have irregular estrous cycles, polycystic ovaries, and disordered hormone levels. (A) The cell types in vaginal smears corresponding to each period. a: proestrus, predominantly consisting of nucleated epithelial cells; b: estrus, with cornfield squamous epithelial cells; c: metestrus, consisting of cornified squamous epithelial cells and predominance of leukocytes; d: diestrus, consisting predominantly of leucocytes (as shown by the arrow). (B) Estrus cycle was examined in control and PCOS groups of mice by evaluating the cell types in vaginal smears. (C) Ovarian and follicular morphology was assessed by H&E staining (2.5x, 5x, and 10x). (D) Linear regression equation shows the relationship between the O.D. values at the wavelength of 450 nm and the concentrations of rat serum T, FSH, and LH. (E) Quantification of enzyme-linked immunosorbent assay of serum in the two groups rats. Three independent experiments were performed with similar results. Data are shown as mean ± SEM. ∗∗P < 0.05. Supplementary Figure 2: PCOS-like rat ovarian dysfunction is improved via a combined curcumin and exercise treatment. (A) Ovarian and follicular morphology was assessed by H&E staining (5x and 10x). (B) The levels of GRP-78 and p-IRE1α were measured with immunohistochemical staining. (C, D) TUNEL analysis of the levels of caspase-12 and cleaved-caspase-3 on ovarian sections. Three independent experiments were performed with similar results. Supplementary Figure 3: DHT induces granulosa cell apoptosis mediated by excessive ER stress resulting in GC dysfunction. (A) Cell viability of granulosa cells after tunicamycin-induced was analyzed by CCK-8 kits. (B) GCs were incubated with Annexin V-FITC and PI. The cells were imaged for apoptosis detection using a FV3000 Olympus microscope. (C, D) The apoptosis factors Bax and cleaved-caspase-3 significantly [file 7382900.f1.docx]

**Supplementary materials**

Supplementary data are available at Oxidative Medicine and Cellular Longevity online.

**Sup-Figure 1**


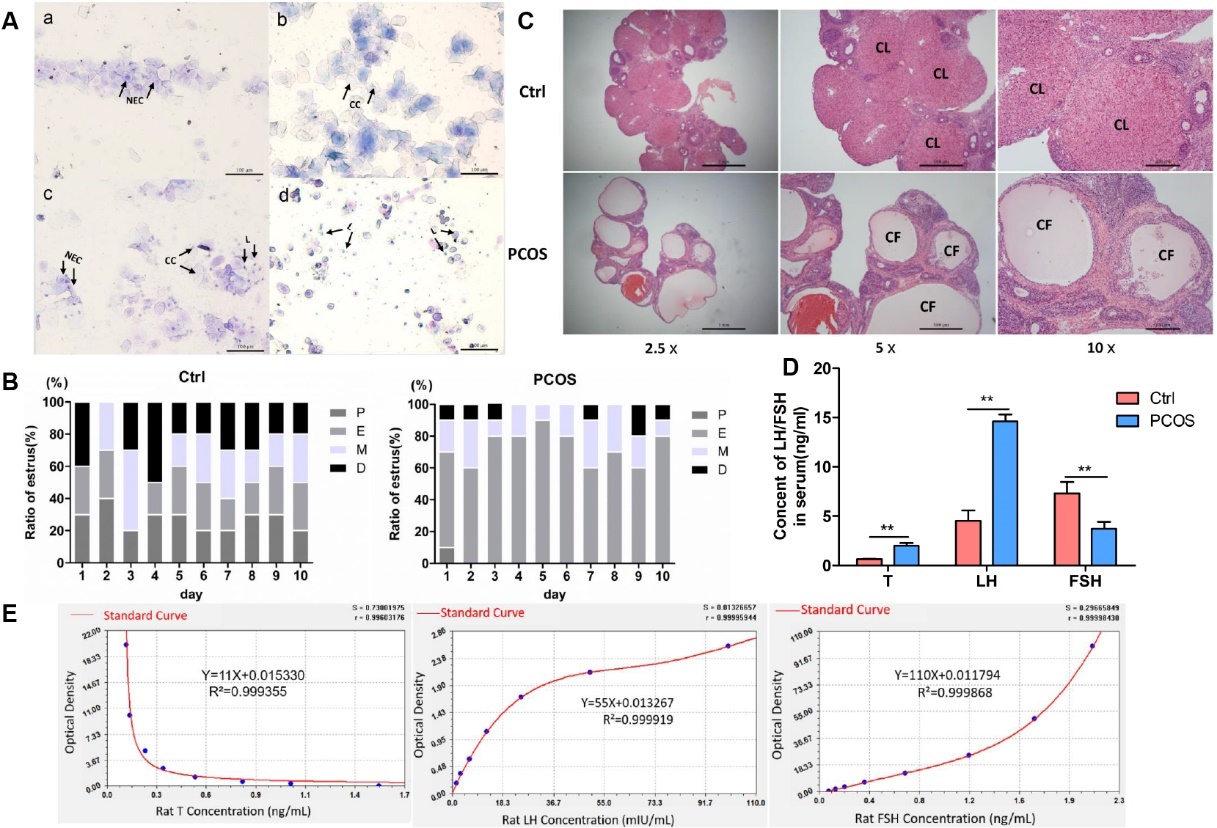


**Sup-Figure 1: DHEA-induced PCOS-like rats have irregular estrous cycles, polycystic ovaries and disordered hormone levels**

Rats were induced with dehydroepiandrosterone-exposed for establish of polycystic ovarian syndrome model. (A) The cell types in vaginal smears corresponding to each period. a: Proestrus, predominantly consisting of nucleated epithelial cells; b: Estrus, with cornfield squamous epithelial cells; c: Metestrus, consisting of cornified squamous epithelial cells and predominance of leukocytes; d: Diestrus, consisting predominantly of leucocytes (As shown by the arrow). (B) Estrus cycle were examined in control and PCOS groups of mice by evaluating the cell types in vaginal smears. (C) Ovarian and follicular morphology was assessed by H&E staining (2.5×, 5×, 10×). (D) Linear regression equation shows the relationship between the O.D. values at the wavelength of 450 nm and the concentrations of rat serum T, FSH, LH. (E) Quantification of enzyme-linked immunosorbent assay of serum in the two groups rats. Three independent experiments were performed with similar results. Data are shown as mean ± SEM. ***P* < 0.05.

**Sup-Figure 2**


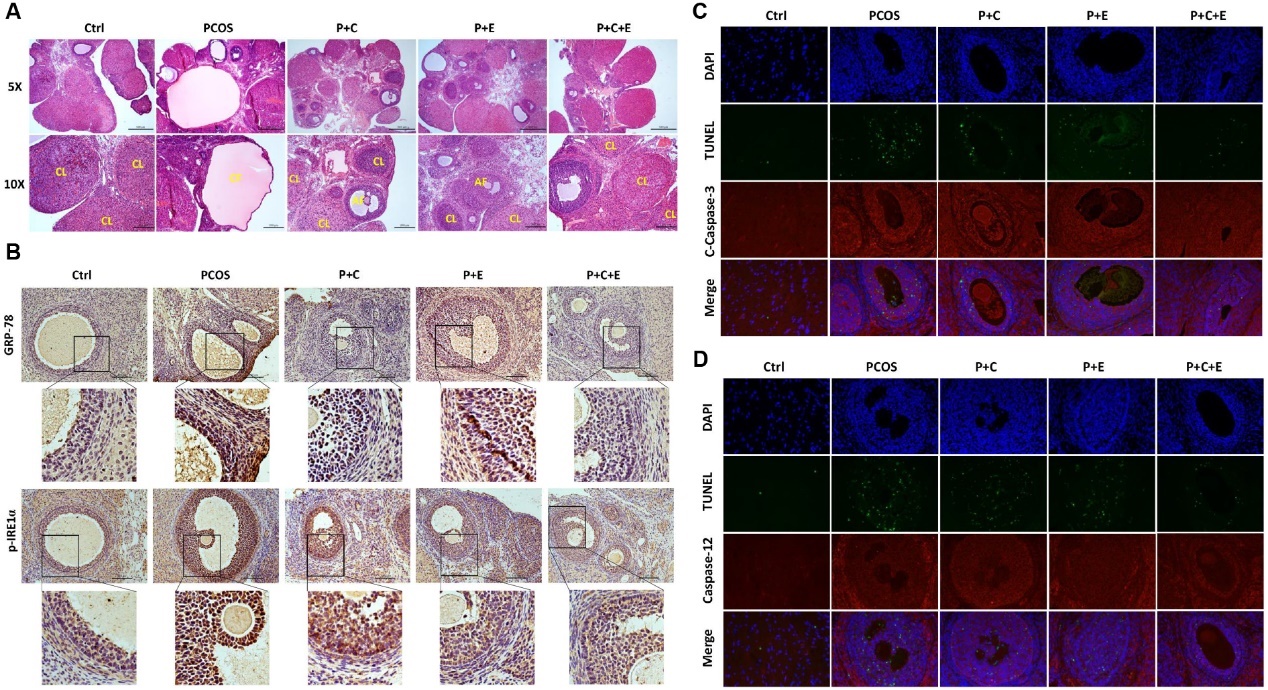


**Sup-Figure 2: PCOS-like rat ovarian dysfunction is improved via a combined curcumin and exercise treatment**

PCOS-like rats received 8 weeks of curcumin gavage, treadmill exercise, and curcumin combined with treadmill exercise treatment. (A) Ovarian and follicular morphology was assessed by H&E staining (5×, 10×). (B) The levels of GRP78 and p-IRE1α were measured with immunohistochemical staining. (C-D) TUNEL analysis the levels of Caspase12 and Cleaved-caspase3 on ovarian sections. Three independent experiments were performed with similar results.

**Sup-Figure 3**

**
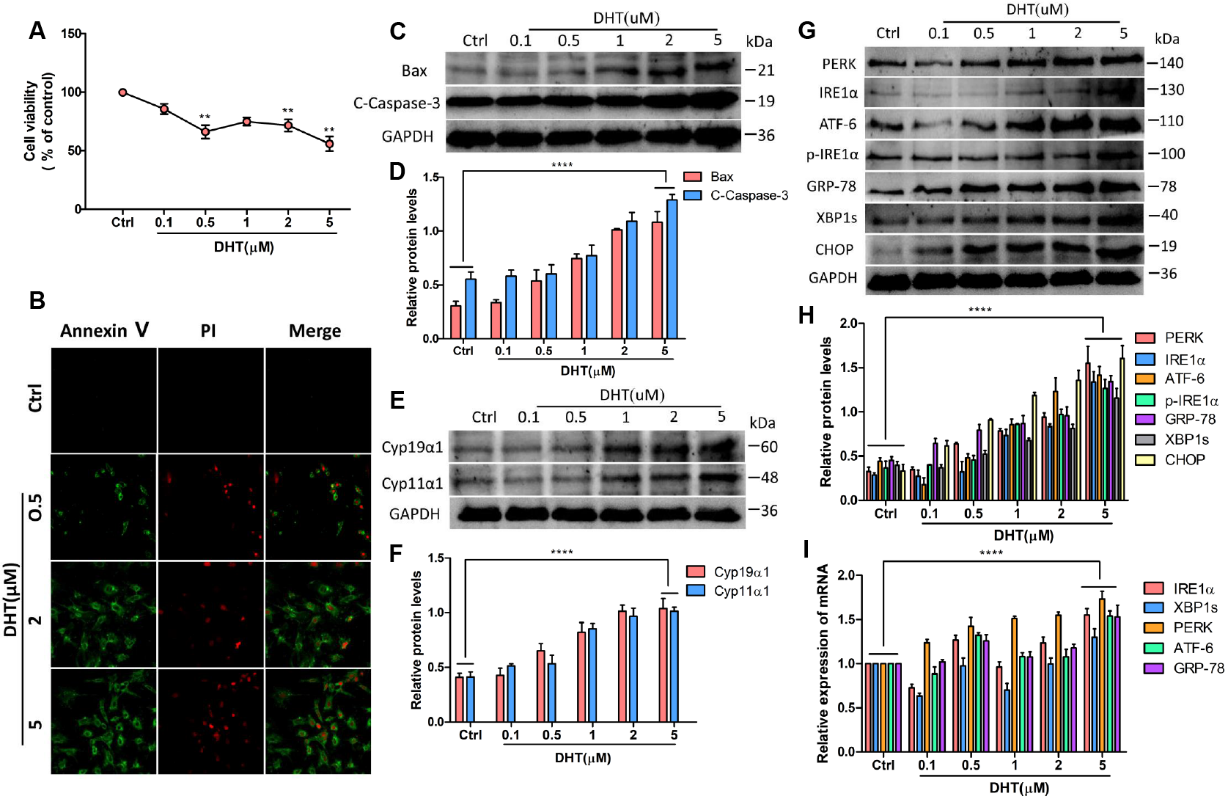
**

**Sup-Figure 3: DHT induces Granulosa cells apoptosis mediated by excessive ER stress resulting in GCs dysfunction**

GCs from naïve SD rats were treated with different concentration of DHT. (A) Cell viability of granulosa cells after t[unicamycin](javascript:;)-inducedwas analyzed by CCK-8 kits. (B) GCs were incubated with annexin V-FITC and PI. The cells were imaged for [apoptosis](javascript:;) detection using a FV3000 Olympus microscope. (C-D) The apoptosis factors Bax and Cleaved-caspase-3 significantly increased by western blot assay. (E-F) The expression of steroid synthase *Cyp11α1* and *Cyp19α1* by western blot assay. (G-I) The protein and mRNA levels of CHOP, GRP-78, ATF-6, PERK, IRE1α, p-IRE1α, XBP1 and Caspase-12 were assessed by western blot assay and qRT-PCR assay. Three independent experiments were performed with similar results. Data are shown as mean ± SEM. ** *P* < 0.05, **** *P* < 0.01.

**Sup-Figure 4**


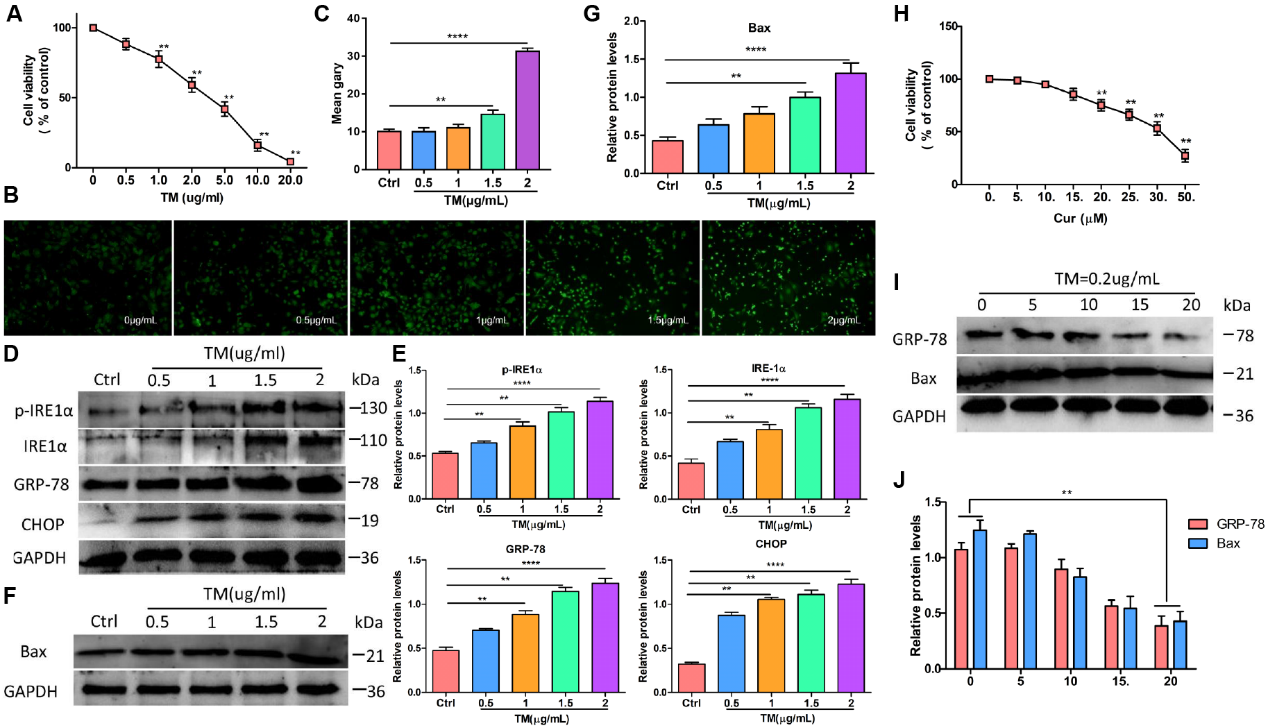


**Sup-Figure 4: Curcumin alleviates tunicamycin (TM)-induced excessive ER stress in GCs, thereby reducing apoptosis**

[Tunicamycin](javascript:;) induced ER stress in GCs. (A) Cell viability of granulosa cells after t[unicamycin](javascript:;)-inducedwas analyzed by CCK-8 kits. (B-C) ROS generation in GCs following[tunicamycin](javascript:;) treatment was measured using the DCF-DA probe. DCF-DA fluorescence (green fluorescence) was measured by confocal microscopy (20×). Images are representative of three independent experiments with similar results. Quantification of the fluorescence is shown. (D-E) The expression of GRP-78, CHOP, IRE1α and p-IRE1α in GCs by different concentration of [tunicamycin](javascript:;) treated by western blot assay. (F-G) The expression level of apoptosis protein Bax was detected by western blot assay. GCs was treated with curcumin in the presence of TM at 0.2μg/mL. (H) Cell viability of GCs after curcumin treated was analyzed by CCK-8 kits. (I-J) The protein level of GRP-78 and Bax in GCs after curcumin treated was analyzed by western blot. (K-L). Three independent experiments were performed with similar results. Data are shown as mean ± SEM. ** *P* < 0.05, **** *P* < 0.01.

**Sup-Figure 5**


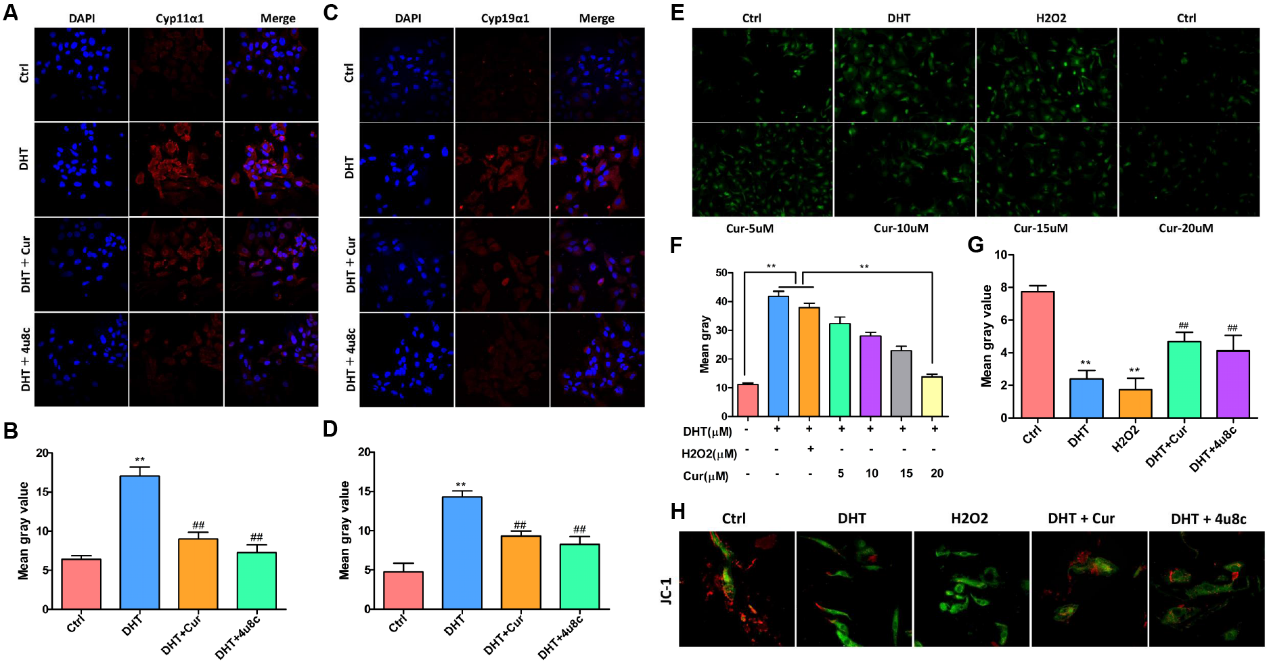


**Sup-Figure 5: Curcumin alleviates DHT-induced ROS and MMP, improved ovarian function**

DHT-induced GCs were treated with different concentrations of curcumin or 4u8c. (A-D) The expression levels of *Cyp11α1* and *Cyp19α1* in GCs with curcumin or 4u8c treated were measured by immunofuorescence staining (60×). (E-F) ROS generation in Granulosa cells following [tunicamycin](javascript:;) treatment was measured using the DCF-DA probe. DCF-DA fluorescence (green fluorescence) was measured by confocal microscopy (20×). Images are representative of three independent experiments with similar results. (G-H) mitochondrial membrane potential was analyzed by the ratio of JC-1 monomers/polymers (60×). Three independent experiments were performed with similar results. Data are shown as mean ± SEM. ** *P* < 0.05. ** *P* < 0.05, vs. Ctrl. ## *P* < 0.05, vs. DHT/H_2_O_2_.
